# Determinants of survival in extremely preterm infants born at 22–23 weeks: a retrospective cohort study

**DOI:** 10.3389/fped.2025.1673565

**Published:** 2025-10-16

**Authors:** Tomonori Kurimoto, Takuya Tokuhisa, Masaya Kibe, Hiroshi Ohashi, Eiji Hirakawa, Takatsugu Maeda, Masato Kamitomo

**Affiliations:** ^1^ Department of Neonatology, Perinatal Medical Center, Kagoshima City Hospital, Kagoshima, Japan; ^2^ Department of Obstetrics and Gynecology, Perinatal Medical Center, Kagoshima City Hospital, Kagoshima, Japan

**Keywords:** extremely preterm infant, 22–23 weeks of gestation, small for gestational age, necrotizing enterocolitis, intraventricular hemorrhage, focal intestinal perforation, tension pneumothorax, cesarean delivery

## Abstract

**Background:**

This study investigated the risk factors associated with mortality in infants born at 22–23 weeks and assessed the impact of different variables on survival outcomes.

**Methods:**

This retrospective study included 185 infants born at 22–23 weeks at a single tertiary care center (2006–2023). Univariate and logistic regression analyses identified independent predictors of mortality.

**Results:**

Among 185 infants, 64 (34.6%) did not survive. Mortality was associated with a lower birth weight (509 g vs. 564 g, *p* = 0.0001) and higher rates of small for gestational age (SGA; 21.9% vs. 7.4%, *p* = 0.009). Independent predictors included SGA [odds ratio (OR): 5.8], tension pneumothorax (OR: 9.9), severe intraventricular hemorrhage (OR: 3.3), focal intestinal perforation (OR: 4.1), necrotizing enterocolitis (OR: 18.9), and early-onset sepsis (OR: 9.7). Cesarean delivery was associated with reduced mortality (OR: 0.3).

**Conclusions:**

Targeted management of these risk factors and cesarean delivery may improve the outcomes in this population.

## Introduction

Advances in perinatal and neonatal care have led to marked improvements in the survival rates of extremely preterm infants born at 22–23 weeks of gestation. In Japan, the survival rates of infants born at 22 and 23 weeks of gestation were reported to be 46.1% and 72.9%, respectively, between 2008 and 2013 (1). More recently, from 2018 to 2020, the survival rates of infants of the same gestational age who underwent resuscitation after birth increased to 63% and 80.0%, respectively (2). Despite these advancements, mortality and long-term morbidity remain substantial challenges for this vulnerable population. Identifying specific risk factors associated with mortality is critical for developing targeted interventions and optimizing outcomes. This study aimed to investigate mortality risk factors in infants born at 22–23 weeks of gestation and evaluate the effects of maternal, neonatal, and perinatal variables on survival rates.

## Materials and methods

### Study design and population

This retrospective cohort study analyzed data from 187 infants born at 22–23 weeks of gestation at a tertiary care center between January 2006 and December 2023. Infants with critical pulmonary stenosis or tetralogy of Fallot were excluded. Of the included infants, 57 (30.8%) were born at 22 weeks, and 128 (69.2%) were born at 23 weeks of gestation. For analysis, the cohort was categorized into two outcome groups: the mortality group (*n* = 64, 34.6%) and the survival group (*n* = 121, 65.4%).

### Gestational age and birth weight

Gestational age and birth weight were determined based on obstetric and neonatal records. The median birth weight of the cohort was 547 g (range: 475–601 g).

### Data collection

Data were collected retrospectively from medical records and included maternal demographics, placental pathology, fetal heart rate (FHR) monitoring results, and neonatal clinical outcomes.

### Definitions

The administration of a complete regimen of antenatal steroids (ANS) was defined as the administration of two doses before delivery in the current pregnancy to promote fetal lung maturation. Abnormal FHR patterns were assessed according to the 2009 recommendations of the National Institute of Child Health and Human Development. Late decelerations were classified as recurrent if they occurred in ≥50% of uterine contractions. Bradycardia was defined as a sustained FHR of <110 bpm for ≥10 min. Infants with birth weights under the 10th percentile were assessed according to standardized Japanese birth size data. Placental pathology, including chorioamnionitis and funisitis, was staged according to the Blanc classification (3). Bacteremia was defined as the detection of microorganisms in blood cultures. Bacteremia identified within the initial 72 h of life was defined as early-onset sepsis (EOS), whereas cases identified beyond 72 h were classified as late-onset sepsis. Head and cardiac ultrasound examinations were performed by neonatologists within the first 24 h of life, three to four times on Day one, three times on Days two and three, and two to three times on Day four. Cardiovascular support, including dopamine, dobutamine, or steroids, was provided based on vital signs and echocardiographic assessments. Hemodynamically significant patent ductus arteriosus (PDA) requiring treatment was confirmed via echocardiography and managed with pharmacotherapy, typically indomethacin until 2017 and ibuprofen thereafter (since 2018) (4).

Intraventricular hemorrhage (IVH) was diagnosed using Volpe's grading system. Meconium-related ileus was diagnosed when a microcolon or small-sized colon extending to the distal ileum was identified, along with a gradual change in the caliber of the ileum. During laparotomy, the proximal ileum was dilated and filled with viscous meconium. In the present study, only surgically treated cases were included for diagnosis, comprising a total of five cases. Focal intestinal perforation (FIP) was diagnosed during surgery and characterized by macroscopic intestinal perforation with a punched-out lesion but without necrosis. Necrotizing enterocolitis (NEC) was diagnosed based on histopathological examination of surgical specimens. Tension pneumothorax was diagnosed based on radiographic findings, including lung displacement away from the chest wall, diaphragmatic depression, mediastinal deviation to the opposite side, or radiolucency along the posterior axillary line on the affected side. Management involved needle aspiration followed by chest tube insertion in all cases. Other forms of air leak, such as pulmonary interstitial emphysema or pneumomediastinum, were not included in this analysis. All infants received empiric broad-spectrum antibiotics (ampicillin and gentamicin) for 5–7 days after birth, in addition to antifungal therapy with micafungin, which was continued until the humidification level decreased to below 80%. The incubator settings were adjusted stepwise: the ambient temperature (T-amb) was first reduced by 0.3°C four times from 37.0°C to 35.8°C, followed by a reduction of the setting humidity (SH) by 5% from 95% to 90%. Thereafter, T-amb was lowered by 0.3°C–35.5°C and SH by another 5%–85%. Finally, T-amb was lowered by an additional 0.3°C and SH by 5% to reach 80% (5). To prevent NEC, oral miconazole was administered for the first 3 weeks of life, in combination with intravenous micafungin (6). Resuscitation measures included the insertion of umbilical arterial and venous lines after birth, fluid volume and electrolyte management, nutrition, and humidity and temperature control in the incubator, following protocols from prior hospital reports (5).

### Ventilation

All infants were intubated in the delivery room due to respiratory distress syndrome or risk of apnea (FiO_2_ ≥ 0.4 to achieve target SpO_2_), followed by surfactant administration. Initial ventilation was provided with synchronized intermittent mandatory ventilation plus pressure support (SIMV + PS). In this mode, the peak inspiratory pressure (PIP) was initially set at 20 cmH₂O and increased up to a maximum of 21 cmH_2_O, and the positive end-expiratory pressure (PEEP) was set at 4.5–6.0 cmH_2_O. Typical settings included a tidal volume of 4–6 ml/kg and inspiratory time 0.3–0.4 s, with continuous monitoring of SpO_2_ and end-tidal CO_2_. If lung aeration was adequate and no atelectasis or air leak was present, infants were transitioned from pressure-limited ventilation to a volume guarantee mode (4–6 ml/kg).

When SIMV failed to improve gas exchange (persistent pH <7.2 and PaCO_2_ > 65 mmHg, or inadequate reduction in PaCO_2_/FiO_2_ within 1 h), high-frequency oscillatory ventilation (HFOV) was initiated as rescue therapy. HFOV was generally set at 12–15 Hz with an I:E ratio of 1:1, and mean airway pressure adjusted 2–3 cmH_2_O above SIMV to achieve lung inflation. The target tidal volume during HFOV was 1.5–2.0 ml/kg.

### Statistical analysis

Univariate analyses were performed to compare variables between the mortality and survival groups using Fisher's exact tests, as appropriate. Statistical significance was defined as a two-tailed *p*-value <0.05. A logistic regression model was constructed to identify independent predictors of mortality. Parameters were estimated using the maximum likelihood method. The outcome variable was mortality, while the predictor variables included small for gestational age (birth weight ≤10%), twin-to-twin transfusion syndrome (TTTS), cesarean delivery, fetal bradycardia, 5 min Apgar score, tension pneumothorax, severe IVH (Grade 3 or 4), FIP, NEC, and EOS. Odds ratios (ORs) with 95% confidence intervals (CIs) were calculated for each predictor. Multicollinearity among independent variables was assessed using the variance inflation factor (VIF), with a threshold of 10 indicating significant multicollinearity. The goodness-of-fit of the logistic regression model was evaluated using the Hosmer-Lemeshow test. Predicted probabilities were grouped into deciles, and the observed and expected frequencies of the outcome were compared using a chi-squared test. A *p*-value >0.05 was considered to indicate adequate model fit. Sensitivity analysis was performed by excluding each predictor variable one at a time to assess the robustness of the logistic regression model. Changes in coefficients, *p*-values, and model fit were compared to the baseline model. Subgroup analyses were also conducted to assess specific interactions and the model's consistency across different clinical contexts.

### Patient and public involvement

Patients or the public were not involved in the design, or conduct, or reporting, or dissemination plans of our research.

## Results

The analysis revealed several significant differences in baseline characteristics between the mortality and survival groups. The median birth weight was significantly lower in the mortality group than in the survival group [509 g, interquartile range (IQR): 435–569.5 g vs. 564 g, IQR: 493–621 g; *p* = 0.0001]. Similarly, SGA was more prevalent in the mortality group than in the survival group (21.9% vs. 7.4%; *p* = 0.0086) (Table 1).

**Table 1:** Risk factors and outcomes in extremely preterm infants (22–23 weeks of gestation).

Characteristics	Survival (% or IQR)	Mortality (% or IQR)	*p*-values
Variables	121 (65.4)	64 (34.6)	
Gestational age (22 GW)	38 (31.4)	19 (29.7)	0.87
Sex (male)	65 (53.7)	31 (48.4)	0.54
Birth weight (median)	564 (493–621)	509 (435–570)	0.0001
SGA	9 (7.4)	14 (21.9)	0.009
MD twin	4 (3.3)	6 (9.4)	0.1
DD twin	22 (18.2)	6 (9.4)	0.13
TTTS	2 (1.7)	6 (9.4)	0.02
Out-of-hospital birth	10 (8.3)	6 (9.4)	0.79
Maternal age	30 (27–34)	31 (28–34)	0.37
Number of pregnancies	2 (1–3)	2 (1–3)	0.36
Number of deliveries	0 (0–1)	1 (0–1)	0.68
Primipara	68 (56.2)	33 (51.6)	0.64
Cesarean delivery	91 (75.2)	37 (57.8)	0.02
Cervical cerclage	6 (5.0)	6 (9.4)	0.35
ACS	36 (29.8)	14 (21.9)	0.30
Tocolysis	87 (71.9)	38 (59.4)	0.10
MgSO4	71 (58.7)	29 (45.3)	0.09
Ritdrine	79 (65.3)	32 (50.0)	0.06
Antibiotics	46 (38.0)	18 (28.1)	0.20
PROM	40 (33.1)	23 (35.9)	0.75
CAM stage 2–3	61 (50.4)	33 (51.6)	1
Funisitis stage 2–3	29 (24.0)	16 (25.0)	0.86
HDP	2 (1.7)	4 (6.3)	0.18
Smoking	1 (0.8)	1 (1.6)	1
GDM	4 (3.3)	0	0.30
HELLP	1 (0.8)	2 (3.1)	0.28
Abruption	14 (11.6)	4 (6.3)	0.30
Fertility treatment (ICSI, IVF-ET, and AIH)	14 (11.6)	7 (10.9)	1
Cephalic presentation	76 (62.8)	39 (60.9)	0.87
Breech presentation	40 (33.1)	19 (29.7)	0.74
NRFS	25 (20.7)	16 (25)	0.67
P/D	7 (5.8)	6 (9.4)	0.38
Severe P/D	4 (3.3)	2 (3.1)	1
V/D	25 (20.7)	14 (21.9)	0.85
Severe V/D	9 (7.4)	9 (14.1)	0.19
L/D	17 (14.0)	6 (9.4)	0.48
recurrent L/D	10 (8.3)	3 (4.7)	0.55
Severe L/D	4 (3.3)	1 (1.6)	0.66
LOV	1 (0.8)	1 (1.6)	1
Bradycardia	3 (2.5)	7 (10.9)	0.03
Tachycardia	5 (4.1)	0	0.17
APS 1 min	3 (1–4)	2 (1–3)	0.25
APS 5 min	6 (5–7)	6 (4–7)	0.007
UApH	7.329 (7.283–7.369)	7.299 (7.183–7.382)	0.23
Tension pneumothorax	9 (7.4)	16 (25.0)	0.001
RDS	121 (100.0)	64 (100.0)	1
IVH grade 3–4	25 (20.7)	30 (46.9)	0.0003
Cystic PVL	5 (4.1)	5 (7.8)	0.32
MRI	3 (2.5)	2 (3.1)	1
FIP	8 (6.6)	14 (21.9)	0.004
NEC	2 (1.7)	13 (20.3)	<0.0001
PDA surgery	13(10.7)	2(3.1)	0.09
EOS	3(2.5)	8(12.5)	0.009
LOS	22(18.2)	18(28.1)	0.14

ACS, antenatal corticosteroids; AIH, artificial insemination with husband; APS, apgar score; CAM, chorioamnionitis; DD twin, dichorionic diamniotic twin; EOS, early-onset sepsis; SGA, small for gestational age (birth weight ≤10%); FIP, focal intestinal perforation; GDM, gestational diabetes mellitus; GW, gestational week; HELLP, hemolysis, elevated liver enzymes, and low platelet count; HDP, hypertensive disorders of pregnancy; ICSI, intracytoplasmic sperm injection; IVF-ET, *in vitro* fertilization and embryo transfer; IVH, intraventricular hemorrhage; L/D, late deceleration; LOS, late-onset sepsis; LOV, loss of variability; MD twin, monochorionic diamniotic twin; MgSO₄, magnesium sulfate; MRI, meconium related ileus; NEC, necrotizing enterocolitis; NRFS, non-reassuring fetal status; PDA, patent ductus arteriosus; P/D, prolonged deceleration; PROM, premature rupture of membranes; PVL, periventricular leukomalacia; RDS, respiratory distress syndrome; TTTS, twin-to-twin transfusion syndrome; UApH, umbilical artery pH; V/D, variable deceleration.

### Definitions

Non-reassuring fetal status (NRFS) is defined as including the absence of accelerations, tachycardia, recurrent late decelerations, severe variable decelerations, severe late decelerations, severe prolonged decelerations, loss of baseline variability, and sustained bradycardia; Severe P/D, Nadir <80 bpm lasting 2–10 min; Severe V/D, Nadir <70 bpm lasting ≥30 s, or nadir 70–79 bpm lasting ≥60 s; Recurrent L/D, Late decelerations occurring with ≥50% of uterine contractions; Severe L/D, Decrease of ≥15 bpm from the baseline to the nadir; LOV, Baseline variability <5 bpm; Bradycardia, Baseline fetal heart rate (FHR) <110 bpm sustained for ≥10 min; Tachycardia, Baseline FHR >160 bpm sustained for ≥10 min.

**Figure 1:**
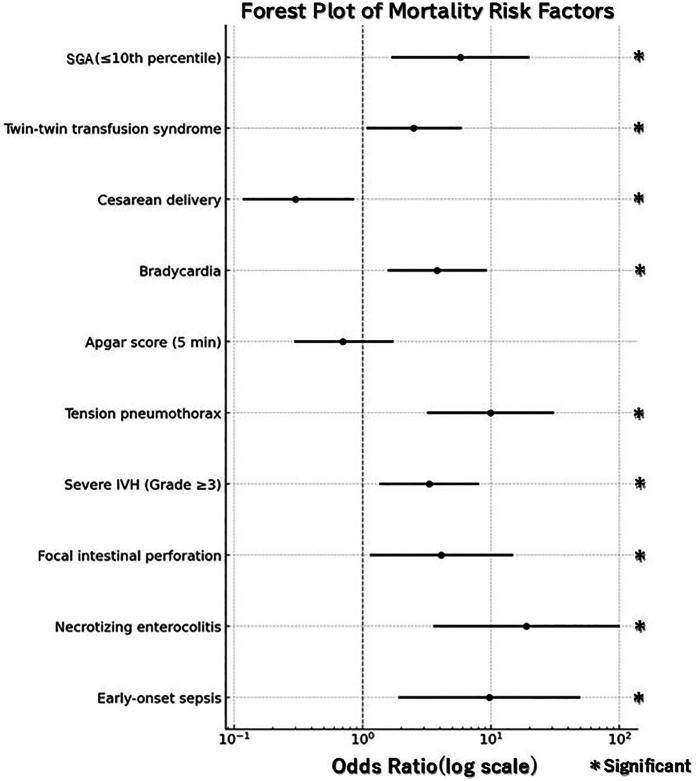
Forest plot for mortality risk factors. Odds ratios (OR) are shown with 95% confidence intervals (CI). Statistical significance was defined as 95% CI not crossing the reference line (OR = 1).

Univariate analyses identified significant differences in multiple variables, including SGA (≤10th percentile), twin-to-twin transfusion syndrome (TTTS), cesarean delivery, fetal bradycardia, 5 min Apgar score, tension pneumothorax, severe IVH (Grade ≥3), FIP, NEC, and EOS. Logistic regression analysis further identified several independent predictors of mortality. SGA (OR: 5.8, 95% CI: 1.72–19.55; *p* = 0.005), tension pneumothorax (OR: 9.9, 95% CI: 3.26–30.24; *p* < 0.0001), severe IVH (Grade ≥3) (OR: 3.3, 95% CI: 1.38–7.91; *p* = 0.007), FIP (OR: 4.1, 95% CI: 1.17–14.60; *p* = 0.03), NEC (OR: 18.9, 95% CI: 3.63–98.77; *p* = 0.001), and EOS (OR: 9.7, 95% CI: 1.94–48.56; *p* = 0.006) were all associated with an increased risk of mortality. In contrast, cesarean delivery demonstrated a protective effect against mortality (OR: 0.3, 95% CI: 0.12–0.84; *p* = 0.02) (Table 2; Figure 1).

**Table 2:** Logistic regression models.

Variables	Odds ratio	95% confidence interval	*p*-value
SGA (10th percentile)	5.8	1.72–19.55	0.005
TTTS	2.9	0.44–18.68	0.27
Cesarean delivery	0.3	0.12–0.84	0.02
Bradycardia	1.8	0.34–9.85	0.48
Apgar score (5 min)	1.3	0.99–1.60	0.052
Tension pneumothorax	9.9	3.26–30.24	<0.0001
IVH (Grade 3–4)	3.3	1.38–7.91	0.007
FIP	4.1	1.17–14.60	0.03
NEC	18.9	3.63–98.77	0.001
EOS	9.7	1.94–48.56	0.006

EOS, early-onset sepsis; SGA, small for gestational age; FIP, focal intestinal perforation; IVH, intraventricular hemorrhage; NEC, necrotizing enterocolitis; TTTS, twin-to-twin transfusion syndrome.

The model's multicollinearity was assessed using variance inflation factor (VIF) analysis, with VIF values ranging from 1.25 to 3.42, indicating no significant multicollinearity (all VIF <10). The Hosmer-Lemeshow test indicated good model fit, with a chi-squared statistic of 4.80, 8 degrees of freedom, and a *p*-value of 0.779. Sensitivity analysis confirmed the robustness of the model. Exclusion of individual predictor variables did not substantially alter the model's overall fit or the coefficients of other predictors. For example, excluding NEC resulted in a minor decrease in pseudo *R*^2^ from 0.325 to 0.318, while other coefficients remained stable. These results demonstrate the model's reliability and its ability to identify significant predictors of mortality in extremely preterm infants.

## Discussion

This study identified key risk factors influencing mortality among infants born at 22–23 weeks of gestation with extreme prematurity. SGA, tension pneumothorax, severe IVH (Grade ≥3), FIP, NEC, and EOS were significantly associated with increased mortality, while cesarean delivery was associated with improved survival.

### SGA

In our study, SGA, defined as a birth weight ≤10th percentile, emerged as a strong predictor of mortality (OR: 5.8, 95% CI: 1.72–19.55; *p* = 0.0046). According to a previous report (7), among infants born at 23 weeks of gestation who received ANS, those with SGA had higher mortality rates compared with those without SGA [relative risk (RR): 1.10, 95% CI: 1.02–1.19]. These findings underscore the significant impact of SGA on survival outcomes in this vulnerable population, even with ANS administration. Currently, there is no consensus on the optimal timing of delivery in pregnancies complicated by early-onset fetal growth restriction. Management must be individualized, incorporating Doppler findings of the umbilical artery and ductus venosus, biophysical profile scores, non-stress test/cardiotocography results, gestational age, and estimated fetal weight (8, 9).

### Tension pneumothorax

Tension pneumothorax, a common complication of mechanical ventilation in extremely preterm infants, was identified as another significant predictor of mortality (OR: 9.9, 95% CI: 3.26–30.24, *p* < 0.0001). In our study, all infants received exogenous surfactant immediately after birth and were managed exclusively with invasive mechanical ventilation; CPAP and non-invasive ventilation were introduced only after extubation, beyond the acute phase. A previous report (10) found that tension pneumothorax occurred in 10.1% of preterm infants born at 22–23 weeks of gestation within the first 72 h after birth. In our study, the incidence was even higher, at 13.5% (25/185), despite the universal use of surfactant and invasive ventilation in the acute phase. This finding underscores the extreme vulnerability of infants at the threshold of viability and suggests that our incidence, although slightly higher, is broadly consistent with earlier reports. Nevertheless, the generalizability of this rate is limited, as different respiratory support strategies, such as early CPAP or non-invasive ventilation, may result in different rates of pneumothorax. HFOV is often used in extremely preterm infants either immediately after birth or within hours of life (11, 12). While systematic reviews suggest that, compared to conventional ventilation, HFOV may slightly reduce the risk of chronic lung disease, it has been associated with an increased risk of acute air leaks (13). Volume guarantee ventilation has been suggested as a strategy for preventing tension pneumothorax. However, its effectiveness in neonates born at 22–23 weeks of gestation remains unclear owing to limited data in this population (14). High-frequency jet ventilation (HFJV) has also been considered in some centers as a potential strategy to reduce the risk of air leaks by delivering very small tidal volumes at rapid rates. However, its use is limited in many countries, including Japan, and robust evidence on its effectiveness in infants born at 22–23 weeks is lacking. The optimal respiratory management for extremely preterm infants born at 22–23 weeks of gestation remains uncertain. Although mechanical ventilation is essential for survival, determining the most effective mode of ventilation remains challenging. After surfactant therapy, unstable lungs often require synchronized ventilation using modes such as SIMV or HFOV. However, the use of advanced ventilatory modes, such as Neurally Adjusted Ventilatory Assist, has not yet been widely adopted or standardized globally. In Japan, SIMV is the most commonly used ventilation mode (2).

Severe IVH grade ≥3

Severe IVH (Grade ≥3) was significantly associated with mortality in this study (OR: 3.3, 95% CI: 1.38–7.91, *p* = 0.0072). Cesarean delivery has been suggested to reduce the risk of severe IVH by minimizing the trauma associated with vaginal delivery. Although a systematic review found no significant association between the mode of delivery and IVH, it did not adequately include infants born at 22–23 weeks of gestation (15). Observational studies of singleton preterm infants (<30 weeks of gestation) have shown that elective cesarean delivery may reduce the risk of IVH in cases of preterm labor (16).

In this study, the administration of a complete course of ANS was independently associated with improved survival and reduced rates of IVH in infants born at 22 0/7–23 6/7 weeks receiving intensive care (17). However, balancing the timing of ANS administration and delivery via cesarean section remains complex. Decisions must consider fetal maturity, risks of delaying delivery, and the need for intervention to optimize outcomes in extremely preterm infants.

### FIP

FIP was identified as a significant risk factor for mortality in this study (OR: 4.14, 95% CI: 1.17–14.60, *p* = 0.027). This finding aligns with previous research indicating that FIP is associated with increased mortality and developmental disabilities in extremely low birth weight infants (18). FIP differs from NEC in its pathophysiology, as it often occurs spontaneously without significant intestinal inflammation. Several factors have been implicated in the development of FIP, including antenatal magnesium or indomethacin exposure, postnatal hydrocortisone or indomethacin exposure, and weight loss ≥15% (19, 20).

### NEC

NEC was the most significant risk factor for mortality in this cohort (OR: 18.9, 95% CI: 3.63–98.77, *p* = 0.0005). This is consistent with the existing literature highlighting the devastating consequences of NEC in extremely preterm infants (21, 22). The risk of NEC is strongly associated with feeding practices. Formula feeding has been associated with a significantly higher risk than does human milk. Therefore, the use of human milk is widely recommended (23). Additional strategies to mitigate NEC risk include minimizing prolonged antibiotic exposure and optimizing the gut microbiome health (24).

### EOS

EOS was another significant predictor of mortality in this study (OR: 9.7, 95% CI: 1.94–48.56, *p* = 0.0056). This finding aligns with those of previous studies linking EOS to increased mortality and neurodevelopmental impairment among extremely preterm infants born between 22 and 26 weeks of gestation (25). Our findings highlight the importance of stringent infection control measures, including strict hand hygiene and prompt administration of antibiotics. However, caution is required with prolonged empirical antibiotic use, as it may increase the risk of NEC (24). Developing rapid diagnostic tools to differentiate true infections from contaminants is crucial to reducing unnecessary antibiotic exposure.

### Cesarean delivery

Cesarean delivery was associated with a significant reduction in mortality (OR: 0.32, 95% CI: 0.12–0.84, *p* = 0.02). This aligns with the findings of previous studies demonstrating improved survival outcomes in extremely preterm infants delivered via cesarean section (26, 27). The protective effect of cesarean delivery may be attributed to the reduced mechanical stress during delivery, resulting in better cardiorespiratory stability at birth. However, decisions regarding cesarean delivery should balance maternal health risks and potential long-term neurodevelopmental outcomes in surviving infants. This finding is particularly notable for maternal–fetal medicine specialists in the United States, where cesarean delivery at 22–23 weeks of gestation remains highly controversial due to concerns about maternal morbidity, implications for future pregnancies, and uncertain neonatal benefit. Highlighting the protective association observed in this study underscores the need for continued dialogue across international perinatal care practices, and calls for further prospective multicenter studies to clarify the role of cesarean delivery at the threshold of viability.

### Limitations

This study has some limitations that warrant careful consideration. First, this study was conducted at a single institution, which may limit the generalizability of the findings to other centers with different clinical practices, patient populations, and demographic characteristics. Multicenter studies are warranted to validate and expand upon our results.

Second, although the sample size of 185 infants was relatively large for retrospective studies focusing on extremely preterm infants born at 22–23 weeks of gestation, it may still be insufficient to detect associations with rare outcomes of risk factors. Despite this, the sample size likely reduced the likelihood of type II errors, particularly for more common outcomes.

Third, owing to the retrospective design of this study, there is a potential for selection and information bias. Nevertheless, the dataset contained no missing values for the extracted variables. However, certain subtle clinical features, undocumented interventions, and environmental factors might not have been comprehensively captured.

Fourth, this study examined associations but did not establish causal relationships between the identified risk factors and mortality. To confirm these findings, prospective studies with controlled designs are required.

Finally, this study reflects on the clinical practices, protocols, and technologies used during the study period. Advances in neonatal care and evolving treatment strategies since then may limit the applicability of these findings to current clinical settings.

Future research should address these limitations through larger multicenter cohort studies, prospective designs, and the use of advanced statistical methods to mitigate biases and account for unmeasured confounders.

## Conclusion

SGA, tension pneumothorax, severe IVH, FIP, NEC, and EOS were identified as risk factors associated to mortality in infants born at 22–23 weeks of gestation. Cesarean delivery demonstrated a significant survival benefit in this population. These findings emphasize the importance of early identification and targeted management of high-risk conditions in extremely preterm infants. Future research should focus on optimizing perinatal and neonatal interventions to address these challenges.

## Data Availability

The dataset presented in this study is available in a public repository. The repository name and accession number(s) can be found in the article/supplementary materials.
